# Teledentistry in the Era of Digital Dentistry: Clinical Applications, Patient Experience, and Equity-Oriented Policy Implications

**DOI:** 10.7759/cureus.100137

**Published:** 2025-12-26

**Authors:** Rawan S Alrehaili, Shahad Almuzaini, Joud Alrajhi, Almiqdad Dashti, Omar Alsuroor, Faisal Alzahrani, Abdulaziz Albishri, Mawaddah Almousa, Lara Homdi, Reem Alshammakhy, Fayafi Alfaifi, Abrar Alkharfi, Ghadeer Aldhafeeri, Thamer M. Alzahrani

**Affiliations:** 1 Dentistry, Private Practice, Medina, SAU; 2 College of Dentistry, Taibah University, Medina, SAU; 3 College of Dentistry, King Saud Bin Abdulaziz University for Health Sciences, Riyadh, SAU; 4 Dental Administration, Ministry of Health, Kuwait City, KWT; 5 College of Dentistry, Imam Abdulrahman Bin Faisal University, Dammam, SAU; 6 College of Dentistry, Vision College, Jeddah, SAU; 7 College of Dentistry, Al-Farabi University, Jeddah, SAU; 8 College of Dentistry, Jazan University, Jazan, SAU; 9 College of Dentistry, Princess Nourah Bint Abdulrahman University, Riyadh, SAU; 10 Periodontology, King Saud Medical City, Riyadh, SAU

**Keywords:** dental monitoring, digital dentistry, remote dental care, remote dental consultation, remote monitoring, teledentistry, telehealth

## Abstract

Teledentistry has shifted from small pilot projects and emergency use during the COVID-19 pandemic to a realistic option for delivering parts of routine oral healthcare. However, its role in long-term service models, equity, and health-system performance remains incompletely defined. This narrative review aimed to provide an up-to-date synthesis of the evidence on teledentistry across clinical specialties, populations, and settings, and to discuss implications for practice, policy, and future research. Electronic databases were searched from inception to December 2025 for studies evaluating teledentistry or related digital remote dental services. Eligible sources included observational and interventional studies, reviews, economic evaluations, implementation reports, and key professional or policy documents. Data were organized thematically around clinical effectiveness, patient and provider experience, cost and resource use, equity and access, regulatory frameworks, and emerging technologies. Across settings, teledentistry supports accurate diagnosis and triage for selected indications, particularly in orthodontics, pediatric dentistry, and community-based screening. Most studies report gains in access, reduced need for travel, and high levels of patient satisfaction, especially in rural, institutionalized, and otherwise underserved groups. Evidence for long-term clinical outcomes, cost-effectiveness, and the impact on oral-health inequities is promising but remains heterogeneous and often limited by small samples, short follow-up, and methodological constraints. Successful programs depend on reliable infrastructure, integration with electronic records, clear clinical protocols, appropriate training, and supportive legal and reimbursement frameworks, while concerns persist about data protection, medico-legal responsibility, and the potential for digital services to widen rather than narrow gaps between population groups. Overall, teledentistry should be viewed as a component of planned hybrid oral healthcare models rather than a substitute for in-person care. Future work needs pragmatic comparative studies, robust economic analyses, and equity-focused evaluations. Under these conditions, teledentistry has the potential to contribute meaningfully to more accessible, continuous, and person-centered oral healthcare.

## Introduction and background

Over the past two decades, rapid advances in information and communication technologies have transformed the delivery of healthcare. Telemedicine, broadly defined as the use of digital communication tools to deliver clinical services at a distance, has progressively evolved from pilot projects for remote communities into an integral component of mainstream medical care in many countries. Dentistry has followed this trajectory with some delay, giving rise to teledentistry, which is the use of telecommunication technologies to provide or support dental care, consultation, education, and public-health activities at a distance [1,2]. Teledentistry includes a wide range of models, from simple telephone triage to real-time video consultations, store-and-forward exchange of digital images, remote monitoring of ongoing treatment, and integration of artificial intelligence (AI) decision support into virtual workflows. In contrast to early initiatives that mainly aimed to connect specialist centers with remote clinics, contemporary teledentistry is embedded in a broader ecosystem of digital dentistry that includes electronic health records, intraoral and extraoral digital imaging, three-dimensional (3D) scanning, computer-aided design and manufacturing (CAD/CAM), and, increasingly, AI-driven diagnostic tools [3].

The COVID-19 pandemic was a major catalyst for the widespread deployment of teledentistry. During periods of lockdown and severe restrictions on elective care, many dental practices adopted remote consultations to maintain contact with patients, provide triage, deliver self-care advice, and prescribe medications where appropriate. Several surveys and observational studies reported that teledentistry helped to sustain essential oral healthcare, reduce unnecessary emergency visits, and support infection control by limiting in-person encounters [4,5]. This exceptional context accelerated adoption and generated a rapidly expanding body of research on the feasibility, acceptability, and short-term outcomes of remote dental care. However, as dentistry transitions from emergency adaptation to long-term integration of digital tools, the role of teledentistry is being reconsidered. Emerging evidence suggests that teledentistry can improve access for underserved populations, such as residents of rural or remote areas, elderly people, individuals with disabilities, and those facing financial or transportation barriers [2,4]. Remote dental screening and triage programs have been implemented in schools, nursing homes, and community settings, often demonstrating high levels of patient satisfaction and potential efficiency gains. At the same time, concerns remain regarding diagnostic accuracy for certain conditions, the quality and continuity of care, medico-legal responsibilities, data security, and the risk that digitalization might inadvertently widen existing inequalities if vulnerable groups have limited access to devices or reliable internet connections [5].

Several reviews have already synthesized aspects of this evolving field. Early narrative and scoping reviews described the basic concepts, technologies, and pilot applications of teledentistry, often emphasizing its potential to improve access and reduce costs [1,5]. More recent overviews have focused on the use of teledentistry during the COVID-19 pandemic, on specific populations or settings, and, in some cases, on particular outcome domains such as knowledge, attitudes, and practices among dental professionals [2,6]. Nevertheless, the literature remains fragmented across specialties and study designs, and important questions about the clinical performance, health-system impact, and sustainable implementation of teledentistry in routine care are still incompletely answered. In parallel, the technological landscape is shifting rapidly. Remote monitoring platforms that combine patient-reported data with images captured on smartphones or intraoral cameras are being evaluated in orthodontics and preventive dentistry. AI-based systems for caries detection, periodontal assessment, and lesion recognition are beginning to be integrated into teleconsultation workflows, raising both new opportunities for earlier detection and new concerns about algorithmic bias, transparency, and accountability. These developments suggest that teledentistry should no longer be viewed only as a temporary alternative to face-to-face encounters but rather as a central component of evolving hybrid care pathways, in which remote and in-person services are strategically combined.

Against this background, there is a need for a comprehensive, critical synthesis that brings together current knowledge on teledentistry, maps its integration into the wider digital dentistry ecosystem, and examines the available evidence on clinical, patient-reported, and system-level outcomes. This comprehensive narrative review aims to address this need by: describing the conceptual foundations and technological modalities of teledentistry within the broader context of digital oral healthcare; synthesizing evidence on clinical applications and outcomes across major dental specialties, including access, diagnostic performance, treatment outcomes, patient and provider experience, and economic implications; discussing ethical, legal, and regulatory issues, with particular attention to equity and the digital divide; and identifying key gaps and outlining future directions, including the integration of AI-enabled tools and other emerging digital technologies. With a broad perspective, this review seeks to inform clinicians, researchers, educators, and policymakers about the current and potential roles of teledentistry in modern oral healthcare and to support evidence-informed decisions on its implementation beyond the pandemic era.

## Review

Search strategy

This review is narrative in nature and does not aim to provide a systematic or exhaustive synthesis of all published studies. Nevertheless, a structured literature search was conducted to identify relevant evidence. From database inception to December 2025, MEDLINE (via PubMed), Embase, Scopus, and Web of Science were searched using combinations of keywords and MeSH terms related to teledentistry (e.g. “teledentistry”, “tele-dentistry”, “remote dental consultation”, “tele-oral medicine”), and digital technologies (e.g. “telemedicine”, “mHealth”, “artificial intelligence”, “remote monitoring”). Reference lists of key articles and recent reviews were screened to identify additional studies. Priority was given to peer-reviewed original research and reviews published in English, with particular emphasis on studies reporting clinical outcomes, patient or provider perspectives, implementation experiences, or health-economic evaluations. Descriptive reports, editorials, and commentaries were considered when they provided important contextual or conceptual information. The literature was synthesized thematically, with the goal of integrating findings from diverse study designs into a coherent narrative.

Conceptual framework and definitions

Teledentistry is commonly defined as the use of telehealth systems and methods in dentistry to deliver care and education at a distance [7]. Policy documents distinguish between synchronous and asynchronous teledentistry, with hybrid models that combine both. Synchronous teledentistry refers to real-time interaction, usually two-way video and audio, between a clinician and a patient, caregiver, or another provider. Asynchronous, or store-and-forward, teledentistry involves capturing images, radiographs, scans, or structured histories and transmitting them for later review and feedback. Teledentistry can appear at different points in the care pathway. It can be used for remote screening and triage before an in-person visit, for diagnostic consultation and treatment planning, and for follow-up and maintenance after procedures. Reviews consistently describe these roles in school programs, long-term care facilities, and community clinics [6,8]. Prevention and health promotion are also frequently used. Fédération Dentaire Internationale (World Dental Federation) (FDI) notes that reminders and educational messages sent via text or apps can support oral hygiene and treatment adherence [9]. Head Start guidance for oral health describes the use of teledentistry to provide preventive care and reduce missed school days in rural and underserved areas.

The stakeholder landscape is wide. Patients and caregivers interact with dental teams but often depend on intermediaries such as teachers, community health workers, or nursing-home staff to capture images or help connect online. The American Academy of Pediatric Dentistry (AAPD) policy and the FDI fact sheet both stress the importance of training these intermediaries and clarifying their responsibilities [9]. An umbrella review on teledentistry and access to care notes that health systems, insurers, and regulators shape reimbursement, licensure, and data-governance rules that can either facilitate or constrain these services [10]. Teledentistry also sits in a broader digital-health ecosystem. The FDI policy statement on digital dentistry describes how digital radiography, intraoral imaging, and 3D scanning form the basis for many tele-services by allowing high-quality data capture and sharing [9]. A recent consensus statement on integrated electronic health records argues that interoperable, patient-centered records that include oral-health data are necessary for safe telehealth and teledentistry [11]. Public-health-oriented documents emphasize that teledentistry can reduce inequalities by providing services to people in rural or conflict-affected settings, as shown, for example, in Sudan during armed conflict. Teledentistry in the time of conflict in Sudan [12]. Teledentistry includes any organized use of digital communication technologies that support or enhance dental care, whether synchronous or asynchronous, direct-to-patient or provider-to-provider. Informal communication, such as personal messaging apps, is only considered when it forms part of a structured care pathway with documentation and clear professional responsibility.

Technological backbone of teledentistry

**Figure 1:**
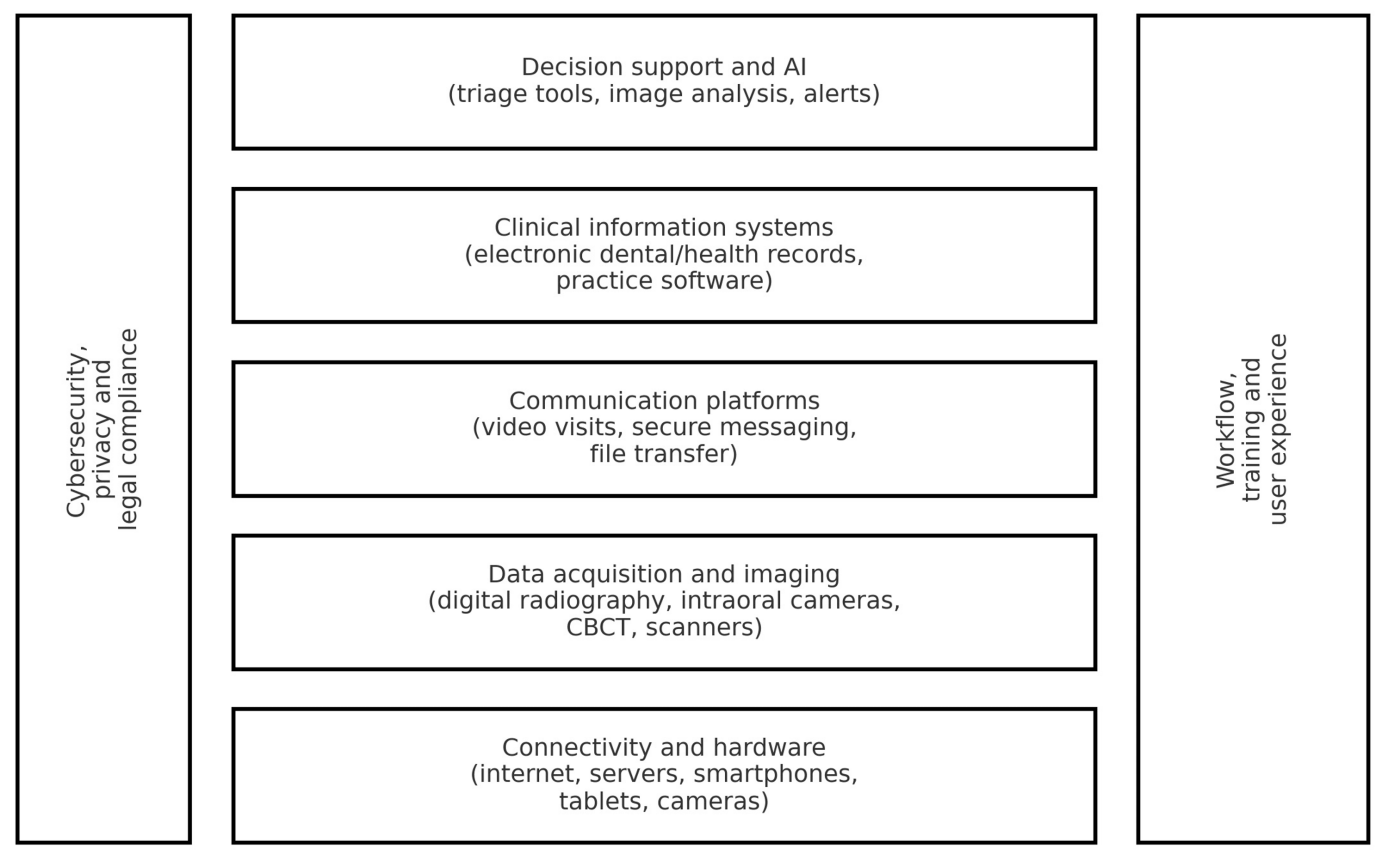
Layered technological architecture underpinning teledentistry CBCT: cone beam computed tomography; AI: artificial intelligence Image credits: Authors

Teledentistry depends on several technological layers. These include devices used to capture clinical information and platforms that allow secure communication and data exchange. Additionally, it includes electronic records that store the information as part of the legal clinical file. Recent reviews and policy statements on teledentistry and digital dentistry emphasize that these components need to work together if services are to be safe, efficient, and sustainable (Figure 1) [6].

Devices and Data Acquisition

Many teledentistry programs use consumer devices such as smartphones or tablets to capture intraoral photographs or short videos. A recent systematic review concluded that photographs taken with digital cameras or smartphones can support caries diagnosis at a distance when image quality is adequate, and examiners follow standardized protocols [13]. Another study from Peru validated a smartphone-based photographic method for diagnosing dental caries in adults and reported good agreement with face-to-face examination [14]. Similar results have been reported for smartphone photographs taken by parents or laypersons in pediatric settings [15]. More advanced models integrate professional imaging devices. Provider-to-provider teledentistry often involves sharing digital radiographs, panoramic images, cone beam CT scans, and intraoral scans for remote interpretation or co-treatment planning. Classic and recent reviews describe such use for oral-medicine consultations, orthodontic treatment planning, and oral-surgery referrals, particularly where access to specialists is limited [6,8]. The FDI also notes the role of high-resolution imaging and intraoral cameras in teleconsultation and remote decision-making [9]. Some programs include peripheral or home-use devices, such as plaque-disclosing tools or simple oral-hygiene monitoring apps. These tools are still emerging, but narrative reports on hybrid care models describe how repeated image capture and remote feedback can support more continuous monitoring rather than episodic visits [6,10].

Communication Platforms and User Interfaces

The communication layer connects patients, caregivers, and dental teams. Early reports often used general videoconferencing tools, but current programs more often rely on dedicated telehealth platforms or teledentistry modules inside practice-management systems. From the patient's perspective, teledentistry is usually accessed through mobile applications or web portals. These interfaces allow users to fill in medical histories, upload images, give consent, join video consultations, and view recommendations. The recent policy emphasizes that platforms should be easy to use and accessible for families with different levels of digital literacy, especially when children or individuals with special health care needs are involved. Implementation reports in children’s programs also note the need for clear instructions and technical support so that caregivers can successfully capture and upload images. Dental teams usually access the same information through clinician dashboards that show demographic data, medical and dental histories, shared images, and consultation notes. Overviews of teledentistry and digital dentistry suggest that integration of these dashboards with existing practice software and electronic records reduces duplicate data entry and helps maintain consistent documentation [9].

Electronic Records and Interoperability

Data generated during teledentistry encounters should be stored in electronic dental records or broader electronic health records as part of the legal clinical file. A recent narrative review on electronic health records in dentistry notes that structured, interoperable, and patient-centered systems are essential for integrating oral and general health data and for supporting telehealth and research [16]. The FDI consensus statement on integrated electronic health records argues that oral-health data should be stored on equal footing with medical data and that interoperable systems are needed to link dental and medical records for early detection, referral, and teleconsultation [11]. A descriptive study from a hospital dental clinic in Indonesia found that the electronic dental record system did not fully meet integrity and non-repudiation requirements, underlining ongoing gaps in record security and completeness [17]. These issues have direct implications for teledentistry. Remote consultations that are not properly documented or are stored outside the main record can compromise continuity of care and medico-legal accountability, as stressed in both electronic health record (EHR) reviews and teledentistry policies [16].

Decision Support

Decision-support tools are closely linked with teledentistry workflows. Some systems use simple rules to guide triage based on reported symptoms. Others use pattern recognition in radiographs or photographs to support the detection of caries or other conditions. A narrative review describes how such tools can assist remote screening, triage, monitoring, and record-keeping, especially when combined with smartphone-based image capture [18].

A scoping review on AI in teledentistry reports that AI models are being integrated into teleconsultation and remote-monitoring platforms to pre-classify incoming images, provide diagnostic suggestions, and flag areas of concern for further review [19]. In the same vein, systematic reviews of AI in dentistry show high technical performance in imaging tasks but emphasize that most studies are retrospective and conducted in controlled settings, with limited validation in routine practice [20,21]. On the other hand, a systematic review focused on AI models for detecting dental caries on oral photographs found high sensitivities and specificities in experimental settings [22]. Yet, it noted limited evidence from prospective clinical use. Another recent review reached similar conclusions for AI-based caries detection on radiographs [23]. Current guidance, therefore, recommends that AI tools in teledentistry be used as aids rather than replacements for clinical judgement and that their performance and fairness be monitored over time [24,25].

Data Protection

Since teledentistry involves the transmission and storage of identifiable health information, cybersecurity and data protection are central. Reviews on electronic health records and health-record security emphasize the need for strong access control, encryption, audit trails, and staff training [16]. A study of dental electronic medical records reported deficiencies in the completeness of records and in security aspects such as integrity and non-repudiation [17]. Professional and regulatory documents on teledentistry, such as FDI, state that teledentistry must meet the same legal and ethical standards as in-person care, including informed consent, privacy protection, and secure storage of data [9]. Cloud-based telehealth platforms add further questions about where data is stored, who is responsible for security, and how long information is retained. General reviews on EHR security underline that clear contracts and governance structures are required to manage these issues [26].

Human and Organizational Factors

The success of teledentistry depends not only on technology but also on how it is adopted by people and organizations. Umbrella and overview reviews on teledentistry report better outcomes when services are integrated into existing workflows, supported by clear protocols, training, and leadership [6,10]. Implementation studies in children’s programs show that reliable internet access, simple image-capture instructions, and alignment with existing reporting systems are important for sustainability [27]. These experiences support the view of teledentistry as a socio-technical system. Devices, software, regulations, and human behavior interact closely, and changes in one element affect the others.

Major dental applications

General Dentistry and Primary Care

In general practice, teledentistry is mainly used for triage, consultation, and short-term follow-up. A narrative review on telemedicine and digital tools in dentistry describes how remote consultations help dentists assess urgency, provide advice, and decide which patients require in-person care, particularly in settings with limited access or during service disruption [28]. During and after the COVID-19 pandemic, cross-sectional studies from several countries reported that dentists used video or telephone consultations to give analgesic and antibiotic prescriptions, provide self-care advice, and postpone non-urgent treatment, while still monitoring pain or swelling [29]. This work showed that many acute problems could be stabilized remotely and that patients valued the convenience of advice without travel. Teledentistry has also been used to improve access and continuity of care in underserved communities. A cluster randomized trial in Iran found that a school-based teledentistry program, which combined remote screening with parental counselling and referral, maintained children’s oral health outcomes at follow-up and was not inferior to conventional chairside examinations [30]. Similar school and community programs in other settings have shown that remote screening can detect caries and refer children for treatment at an early stage [31]. More broadly, a narrative review on teledentistry and oral health outcomes concluded that teleconsultations and remote advice can reduce travelling time, improve attendance at follow-up appointments, and support time- and cost-efficient management of common problems in primary care.

Orthodontics

Orthodontics is one of the most active fields for teledentistry, particularly for remote monitoring of fixed appliances and clear aligners. A systematic review and meta-analysis concluded that teledentistry using Dental Monitoring® software (Dental Mind, Paris, France) can be an effective aid in monitoring orthodontic treatment, especially with clear aligners. The review found a significant reduction in the number of face-to-face visits and shorter time to start refinement, although the certainty of evidence was low to moderate [32]. Building on this, a systematic review on the effectiveness of Dental Monitoring® in orthodontics reported that integrating this remote-monitoring system into routine care reduces in-office appointments and can maintain treatment quality, but highlighted heterogeneity in study designs and the need for more robust trials [33]. Additionally, another review of tele-orthodontics and sensor-based technologies found that remote platforms can help monitor elastic wear, aligner fit, and appliance breakages, and may improve patient compliance through more frequent feedback [34]. 

Clinical studies support these findings. A recent article on digital orthodontic treatment monitoring reported that remote assessments of photographs and scans could identify issues early, reduce unnecessary visits, and increase efficiency without compromising outcomes [35]. A survey of orthodontists showed that adoption of remote-monitoring platforms increased markedly during the COVID-19 pandemic and remained higher afterwards, although clinicians varied in how strongly they integrated these tools into routine care [36]. Patient and clinician perspectives are generally favorable but not without concerns. A 2021 study on attitudes toward Dental Monitoring® reported that many orthodontists and patients appreciated the convenience and perceived closer supervision, while some raised questions about workload, data security, and the risk of over-monitoring [37]. Overall, the evidence suggests that teledentistry is a useful adjunct in orthodontic care for screening, monitoring, and follow-up, especially in aligner therapy, but long-term comparative data on treatment outcomes and stability are still limited.

Pediatric Dentistry

Teledentistry has been widely applied in pediatric dentistry for oral-health education, screening, diagnosis, and behavior support. A narrative review from 2021 reported that teledentistry in children has been used for remote diagnosis of caries and developmental defects, monitoring of treatment, reinforcement of preventive advice, and management of anxious or uncooperative patients [38]. Several studies have evaluated the accuracy of remote caries detection in schoolchildren. Early work using intraoral cameras and telehealth communication in preschool settings found that remote screenings were comparable to visual examinations for identifying early childhood caries [39]. Another study using a teledentistry-based school program showed that digital images captured in classrooms allowed reliable screening for early childhood caries, with high intra- and inter-examiner agreement [31]. More recent work has confirmed and extended these findings. A recent study reported that teledentistry had acceptable accuracy for caries detection in schoolchildren compared with clinical examination [40]. Another diagnostic-accuracy study concluded that teledentistry was a viable alternative to chairside examination for caries screening in schools [41]. Furthermore, a study showed that mobile-phone photographs taken in schools provided caries detection comparable to conventional examination, and highlighted cost-effectiveness and improved access [42].

Beyond diagnosis, pediatric programs have used teledentistry for parental counselling and preventive follow-up. A cluster trial showed that a school-based teledentistry model combining remote screening, education, and referral improved access to care and maintained children’s oral health over time [30]. The AAPD policy on teledentistry notes that these approaches can be particularly valuable for children with special health care needs, because they reduce travel burdens, allow multidisciplinary input, and provide opportunities for desensitization and behavior guidance before face-to-face visits.

Oral Surgery and Oral Medicine

In oral medicine, teledentistry is used mainly for telediagnosis and teleconsultation of mucosal and soft-tissue lesions. A recent study found that teledentistry using digital photographs could provide a reliable diagnosis of oral potentially malignant disorders when compared with clinical examination, although some lesions still required in-person assessment [43]. An earlier study on telediagnosis of oral lesions in primary care reported acceptable diagnostic accuracy when photographs from health centers were assessed by specialists, supporting the use of teleconsultation for triage and early detection [44]. A web-based tool for oral-mucosa teleconsultation showed good usability and reliability, and confirmed that telediagnosis and teleconsultation of oral mucosal lesions are among the most common forms of teledentistry in oral medicine [45]. A recent review concluded that teledentistry could be a useful solution for the diagnosis of oral lesions, particularly by promoting earlier detection in primary care and rural settings, but cautioned that image quality, standardized protocols, and training are crucial [46]. In oral surgery, teledentistry is used for pre-operative assessment, consent discussions, and post-operative follow-up. A recent review on restorative dentistry and related surgical care described how remote consultations can support assessment of pain, swelling, and healing after procedures, and how sharing images allows clinicians to monitor complications and adjust instructions without requiring an in-person visit [47]. Observational reports during the COVID-19 pandemic similarly showed that teleconsultations were useful for monitoring surgical patients, reducing unnecessary emergency visits, and reassuring patients about normal healing [29].

Periodontics and Restorative Dentistry

Evidence for teledentistry in periodontology and restorative dentistry is growing, but remains less extensive than in orthodontics or pediatrics. The 2025 review on restorative dentistry and teledentistry reported that remote imaging and consultations can be used to evaluate periodontal conditions, including probing depths and gingival changes, and that remote assessments were in some studies comparable to in-person evaluation [47]. Teledentistry has been proposed to support maintenance of periodontal and peri-implant health through remote monitoring of plaque, inflammation, and oral hygiene, although most of the literature focuses on general diagnostic measures rather than tele-specific trials. A review on diagnostic measures for lifelong management of periodontal and peri-implant disease highlighted the central role of regular data collection and imaging, which in principle can be adapted to remote settings, but did not yet find strong outcome data from dedicated teledentistry interventions [48]. More generally, digital dentistry and telemedicine reviews note that remote consultations can be used to review radiographs, photographs, and scans related to restorative and implant cases, to discuss options with patients, and to provide structured follow-up after prosthetic or implant treatment [28]. The available evidence suggests feasibility and patient acceptance but does not yet provide strong comparative data on long-term periodontal or restorative outcomes [47].

Special Care Dentistry

Teledentistry has particular relevance for people who face barriers to in-person care, including those in remote areas, conflict zones, long-term care facilities, and communities with limited resources. A recent review found that teledentistry improved early detection of lesions and access to dental care in remote regions and could save time and costs for both patients and providers [4]. School-based programs in different countries show that teledentistry can provide screening and preventive support to children in rural and underserved areas where dental services are scarce [31]. These initiatives often rely on teachers or community health workers to collect images and data, which are then reviewed by dentists. In conflict settings, teledentistry has been used to maintain some level of access when conventional services are disrupted. A report from Sudan described how teleconsultations helped triage urgent cases, advise local providers, and support continuity of care during armed conflict, despite important limitations in connectivity and infrastructure [12]. For older adults and people with disabilities, teleconsultations can reduce the need for travel, allow assessment in familiar settings, and support caregivers with timely advice. The FDI teledentistry fact sheet and several narrative reviews highlight nursing homes and home-care services as key areas where teledentistry can help detect problems earlier and coordinate services with community dentists [9,10]. Overall, across specialties, the literature suggests that teledentistry can enhance access, support early detection, and improve convenience, especially when used as part of structured programs rather than as isolated encounters. At the same time, the strength of evidence varies between fields, with orthodontics and pediatric dentistry screening being more extensively studied than periodontal or implant maintenance, and with limited long-term comparative data for many outcomes.

Evidence on outcomes

Access, Utilization, and Equity

Across multiple reviews, teledentistry consistently improves access to oral health care for people who live far from clinics or face mobility and financial barriers. An umbrella review of 22 systematic reviews concluded that teledentistry interventions usually increased access, reduced travel and waiting time, and supported continuity of care, although the overall certainty of the evidence was low to moderate [49]. A recent systematic review of teledentistry in rural and underserved populations reported that in eight primary studies, remote models significantly improved access to dental care, with high diagnostic concordance (often 85%-90%) between remote and face-to-face examinations and substantial reductions in travel burden [50]. Another umbrella review focused on oral outcomes. Al-Buhaisi and colleagues reported that teledentistry enhanced early detection of oral lesions and increased access to care in remote areas, with many studies describing time and cost savings, though heterogeneity and risk of bias limited firm conclusions [4].

Diagnostic Accuracy and Clinical Effectiveness

A systematic review found that teledentistry achieved diagnostic accuracy for several conditions (including dental caries and orthodontic problems) that was broadly comparable to conventional examinations, especially when high-quality images or live video were used [51]. Earlier, another study reviewed primary studies on the validity of teledentistry and concluded that remote assessments showed good agreement with in-person diagnoses for caries, periodontal conditions, malocclusion, and oral lesions, although most data came from small, single-center studies [52]. More recent specialty-specific reviews reinforce these findings. A systematic review on caries screening using smartphone or intra-oral camera images reported sensitivities and specificities that were generally in the moderate-to-high range compared with clinical gold standards, supporting the use of remote caries screening in school children and community settings [13]. A diagnostic meta-analysis on oral mucosal lesions concluded that teledentistry was a promising approach for triaging and follow-up of potentially malignant and benign lesions, with good diagnostic performance but considerable variation in study methods and image quality [6]. Furthermore, an umbrella review also noted that although most reviews reported positive effects on clinical outcomes such as plaque scores, lesion detection, or treatment completion, the underlying trials were often small, followed participants for short periods, and used heterogeneous outcome measures, which makes it difficult to quantify effect sizes with precision [49].

Patient-Reported Outcomes and Satisfaction

Patient satisfaction is one of the most consistently positive outcome domains for teledentistry. A systematic review of patient satisfaction with e-oral health in rural and remote settings found that, across interventional and observational studies, e-oral health interventions were associated with higher satisfaction than conventional care and were considered effective and reliable by patients [53]. The same review reported that remote care often reduced waiting time, number of visits, travel demands, and overall costs from the patient perspective, although the authors emphasized that methodological inconsistencies and short follow-up limited the strength of the evidence. In a pilot trial from a temporomandibular disorder clinic, a study compared conventional follow-up visits with Zoom-based teledentistry consultations. They found no significant differences in overall patient experience or satisfaction between the two visit types, and patients reported that virtual visits saved time and travel and could be integrated into routine clinic flow without compromising perceived quality of care [54]. An overview of reviews on access and quality summarized eight systematic reviews that reported patient satisfaction outcomes and concluded that satisfaction with teledentistry was generally high, especially when services reduced travel and waiting time and when technical problems were minimal [2].

Provider Perspectives, Workload, and Implementation Experience

A systematic review and meta-analysis synthesized data on awareness, knowledge, attitude, and practice of teledentistry among dental practitioners during the COVID-19 pandemic [55]. They found generally positive attitudes and high perceived usefulness, but also identified gaps in formal training and concerns about technical reliability, medico-legal responsibility, and reimbursement. Several cross-sectional surveys in different regions have shown that most dentists recognize the value of teledentistry in improving access and supporting consultation with specialists, yet many feel insufficiently prepared to implement it in daily practice because of limited training, unclear guidelines, and lack of organizational support [56]. A study explored challenges and mitigation strategies for teledentistry in Pakistan and highlighted personnel barriers (limited training and resistance to change), technological barriers (infrastructure, software, and connectivity), and organizational barriers (uncertain reimbursement and regulatory frameworks). The authors recommended structured institutional support, clear regulations, and targeted training as prerequisites for sustainable implementation [57]. While many clinicians valued the ability to consult specialists, monitor orthodontic or periodontal treatment, and triage emergencies remotely, they were concerned about additional workload, lack of time for training, and the need for secure documentation and integration with existing electronic records.

Economic Outcomes and Cost-Effectiveness

**Figure 2:**
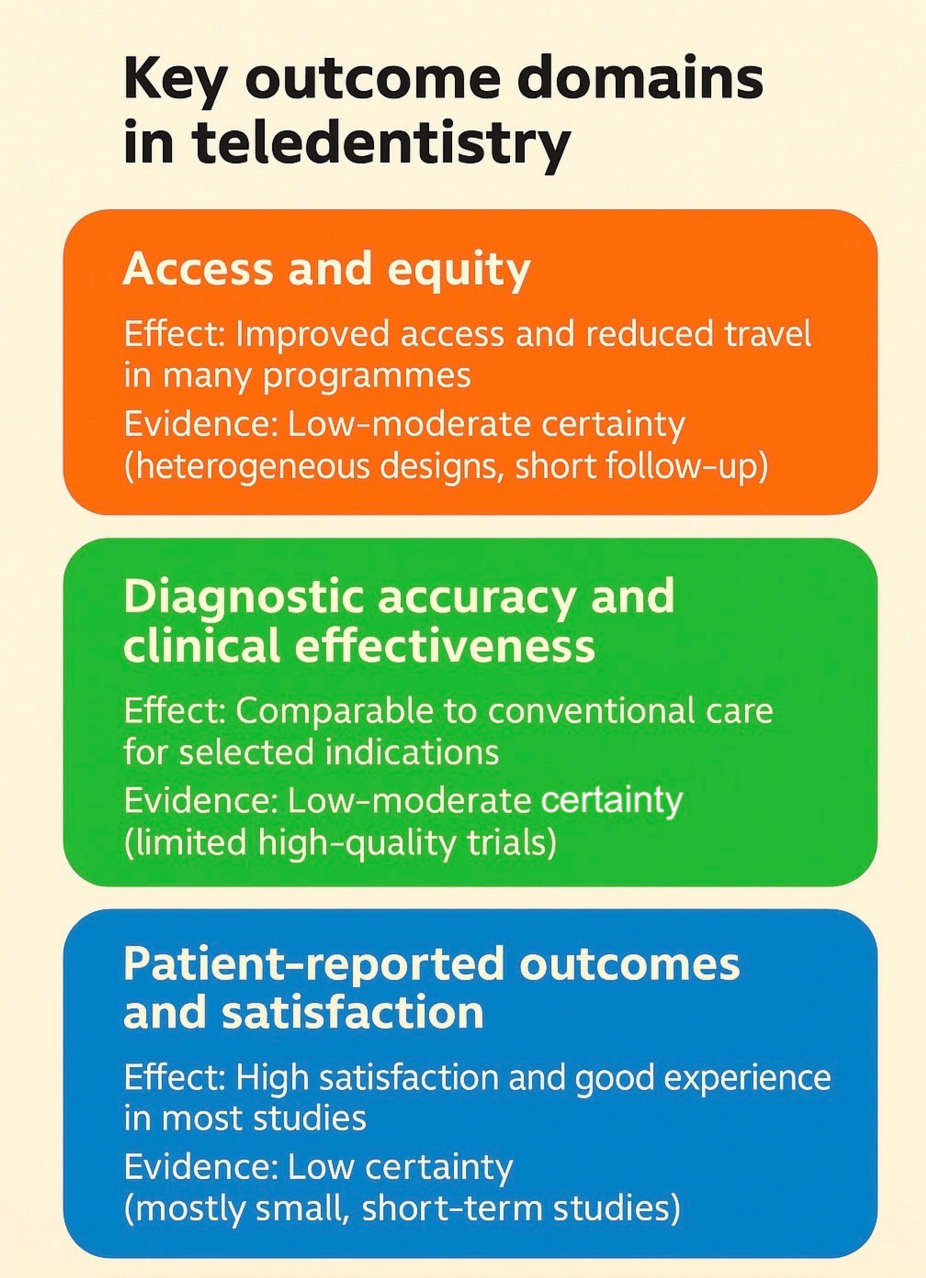
Overview of key outcome domains and certainty of evidence in teledentistry Image credits: Authors

Economic findings are promising but still relatively sparse. An umbrella review identified 10 reviews that reported on costs and economic evaluations, and most concluded that teledentistry can be cost-saving or cost-minimizing compared with usual care; however, the authors stressed that many conclusions were based on modelling assumptions or small trial data rather than robust, long-term cost-effectiveness analyses [49]. Another landmark cost-minimization study compared a statewide school dental screening program delivered by traditional chairside examinations versus a teledentistry model in which mid-level dental practitioners captured images for remote assessment. The teledentistry model was estimated to save about AUD 85 million per year for 2.7 million children by reducing staff salaries, travel, and accommodation costs, while maintaining comparable screening performance [58]. In residential aged care facilities, a study showed that a teledentistry program could reduce the costs associated with transporting frail older adults to dental clinics, suggesting that remote consultation and triage may be economically favorable when travel is difficult, and care needs are frequent [59]. Another economic evaluation of the Highlands and Islands teledentistry project in Scotland compared remote video consultations with outreach specialist visits and hospital referrals. The analysis suggested that teledentistry could become cost-effective as utilization increases and equipment costs are spread over more consultations, particularly when the alternative involves repeated long-distance travel for either specialists or patients (Figure 2) [60].

Limitations of the Current Evidence

Despite encouraging findings, the evidence base still has important gaps. The umbrella review of teledentistry effects highlighted that many primary studies had small sample sizes, non-randomized designs, short follow-up, and high or unclear risk of bias, which limits the certainty of estimates for both clinical and economic outcomes [49]. An overview of systematic reviews also emphasized substantial heterogeneity in teledentistry modalities, populations, comparators, and outcome measures, making meta-analysis difficult and reducing the strength of pooled conclusions [2]. Several recent reviews on underserved populations and rural communities have noted that most studies come from high- or upper-middle-income countries, with limited evidence from low-income settings, and that equity outcomes such as reduction in oral-health inequalities are rarely measured directly [50]. Overall, current data suggest that teledentistry can improve access, achieve diagnostic accuracy close to conventional care, generate high patient satisfaction, and be cost-saving in specific scenarios. However, high-quality randomized trials, standardized outcome sets, and rigorous economic evaluations are still needed before definitive statements about long-term effectiveness and value for money can be made.

Barriers, ethical and legal issues, and future directions

Barriers, Challenges, and Risks

Although the evidence base shows that teledentistry can improve access, communication, and patient-reported experience, several overviews stress that its real-world impact is limited by multilevel barriers. A recent umbrella review highlighted infrastructure, workforce, organizational, and policy constraints as key obstacles to wider adoption of teledentistry [2]. At the system level, reliable internet connectivity, appropriate hardware, secure platforms, and interoperability with electronic health records are not guaranteed, especially in rural or under-resourced regions. Surveys of dentists in European contexts report that weak digital infrastructure, lack of integration with existing practice-management software, and concerns about cybersecurity reduce willingness to rely on virtual care [61]. Studies also show that organizational readiness is uneven. Dental teams describe uncertainty about workflows, scheduling, and triage protocols, and they often lack protected time for remote consultations. Cross-sectional surveys from different regions show that many dentists recognize the value of teledentistry but feel unsure about their technical skills, the convenience of platforms, and compatibility with existing systems. Acceptance tends to be higher among clinicians with postgraduate training and in academic or larger organizational settings, suggesting that experience and institutional support matter [62,63].

On the patient side, digital literacy, access to smartphones or computers, language barriers, and trust in remote care strongly influence uptake. Best-practice reports from public health programs in the United States emphasize that communities facing structural disadvantage, rural residents, people with disabilities, and those with low income may benefit the most from teledentistry, but also face the greatest connectivity and skills gaps. Clinical limitations remain another recurring concern. Research notes that image quality, lighting, and framing are variable, and that remote assessment cannot fully replace tactile examination, periodontal probing, or palpation. Studies suggest that diagnostic accuracy can be high for selected uses, but they also warn about misclassification when images are suboptimal or when complex conditions are evaluated remotely. Economic and policy barriers are equally important. Classic and recent reviews identify uncertain reimbursement, fragmented billing rules, start-up costs for equipment, and a lack of clarity about paying for asynchronous services as major obstacles for clinics. In the United States, reports underline that variability in Medicaid and private-payer policies makes it difficult for practices to plan sustainable teledentistry services, even when clinical value is clear. These findings show that teledentistry is not a stand-alone technology problem. It is a service-delivery change that depends on infrastructure, staffing, training, reimbursement, and thoughtful case selection. Addressing these barriers requires coordinated action by health systems, professional bodies, regulators, and technology vendors, rather than individual clinicians working in isolation [6].

Ethical, Legal, and Regulatory Considerations

Teledentistry inherits the general ethical issues of telehealth and adds some sector-specific challenges. A scoping review on legal issues in digital oral health shows that privacy, data security, cross-border care, informed consent, and professional liability are recurrent themes across jurisdictions [64]. Data protection and confidentiality are central. Narrative reviews focusing on teledentistry and digital oral health emphasize that images, radiographs, and video consultations must be transmitted and stored using encrypted channels, with access controls comparable to or stricter than those for conventional records. They also highlight the need for explicit consent when consultations are recorded, when images are reused for teaching, or when data leaves the country [65,66]. Licensure, scope of practice, and jurisdictional rules are another core area. The American Dental Association policy on teledentistry states that dentists and allied personnel who deliver virtual services must be licensed in the jurisdiction where the patient receives care, and that teledentistry cannot be used to expand the legal scope of auxiliary staff. State-level analyses from the United States show wide variation in how teledentistry is defined, which provider types may deliver it, where patients may be located, and which modalities (synchronous versus asynchronous) are eligible for payment. Reimbursement policies add another legal and ethical layer, because they shape who can actually benefit from remote services. Reports from Medicaid, the US National Conference of State Legislatures, and federal telehealth guidance confirm that states have wide discretion to decide which dental telehealth services are covered, under what conditions, and at what rate. Where teledentistry is reimbursed at lower rates, or only for limited codes, adoption tends to lag, which can widen oral-health gaps between regions. Standard of care and professional liability need careful consideration. Ethico-legal reviews note that dentists remain responsible for clinical decisions made via teledentistry and must ensure that history-taking, documentation, imaging, and follow-up meet the same or higher standard as in-person care. Where physical examination is essential, they recommend that teledentistry be framed as an adjunct for triage, counselling, or monitoring rather than a full substitute for clinical contact [66].

**Figure 3:**
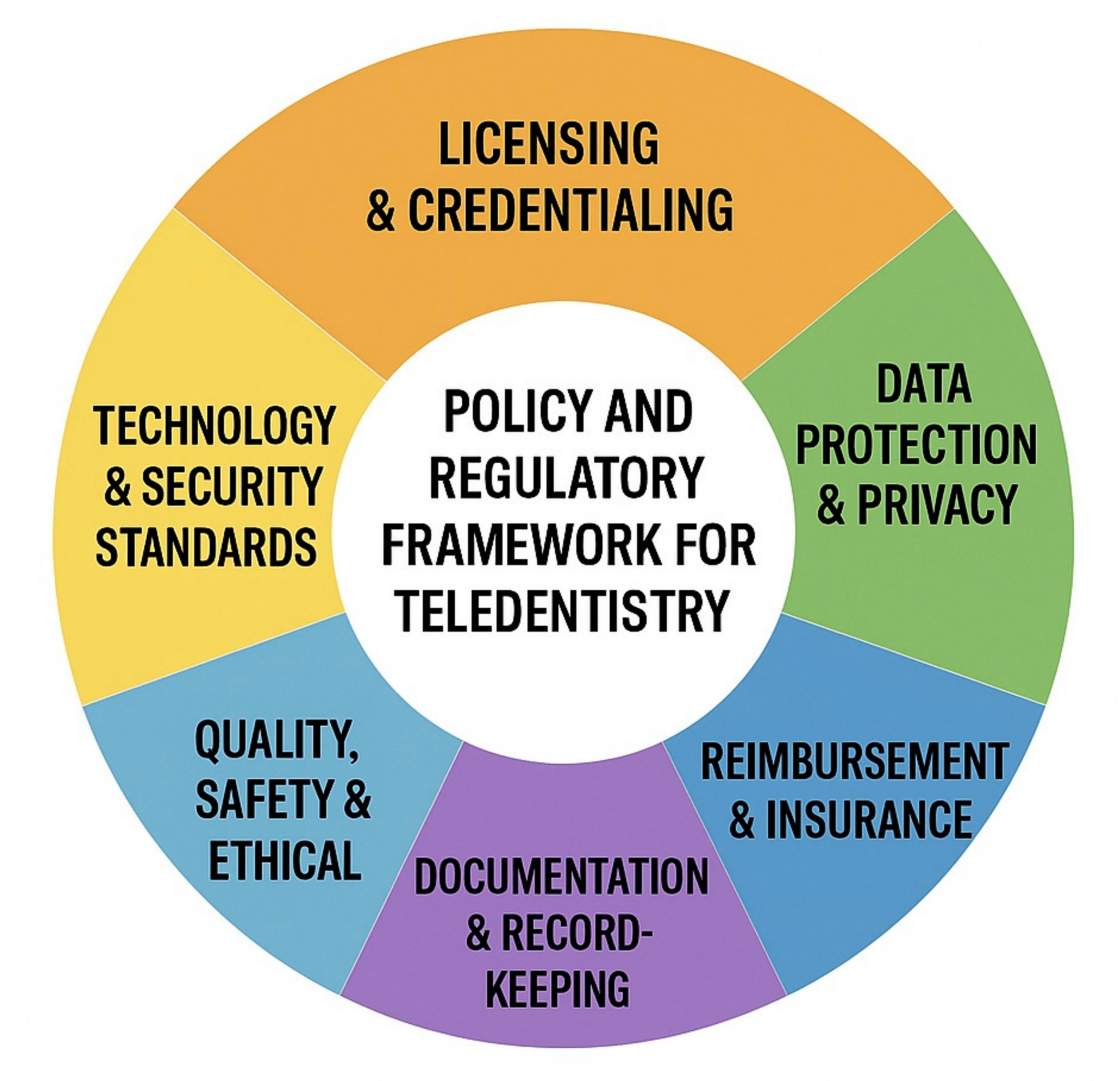
Policy and regulatory framework for teledentistry Image credits: Authors

Equity and justice are also highlighted. It should be noted that if broadband access, device ownership, and digital literacy are not actively addressed, teledentistry may end up serving those who are already relatively advantaged. Policy briefs on oral health for people with special health-care needs, adults with intellectual and developmental disabilities, and rural communities stress that program design must include tailored support such as community facilitators, simple interfaces, and alternative low-bandwidth options. Recent legal and ethical analyses converge on the idea that clear national and regional frameworks, codes of conduct, and practical guidance for clinicians are needed. These should cover consent templates, documentation standards, platform requirements, cross-border arrangements, and responsibilities when technology fails. Such frameworks are beginning to appear in many countries, but there is still marked variation in maturity across regions (Figure 3) [67].

Future Directions

Most recent evidence suggests that teledentistry is moving from pilot projects toward integration in wider digital-health ecosystems. One important direction is the careful use of AI within teledentistry platforms. Hybrid models that combine virtual screening with targeted in-person care are another clear trend. Evidence describes “virtual dental homes,” school-based screening programs, and community outreach models where asynchronous image collection, AI-assisted triage, and subsequent chairside treatment are linked in a single care pathway.

A recent editorial frames this as part of a broader strategy to use teledentistry and AI to strengthen access for vulnerable groups, while also calling for stronger evaluation of outcomes and unintended effects [68]. From a technical perspective, this review anticipates that 5G networks, low-cost smartphones, extended-reality interfaces, and connected oral-health devices will expand what can be done at a distance. Concept articles argue that combining high-resolution imaging, secure cloud infrastructures, and edge computing could allow near real-time remote monitoring of orthodontic treatment, implant healing, or mucosal lesions, provided that governance and reimbursement mechanisms keep pace. Many existing studies have small samples, short follow-up, single-center designs, and heterogeneous outcome measures, which limit meta-analysis and generalization. Recent scoping and umbrella reviews call for pragmatic trials and implementation studies that compare different teledentistry models, evaluate long-term outcomes, and incorporate cost-effectiveness and equity analyses rather than focusing only on diagnostic accuracy. Finally, there is a strong call to keep equity at the center of future work. Policy briefs and public-health reports emphasize designing teledentistry services in partnership with communities, adapting interfaces for people with disabilities, and measuring impact on disparities, not just on average outcomes [69,70]. For example, national reports on adults with intellectual and developmental disabilities in the United States describe how teledentistry might reduce travel burden and expand access, but only if support staff, training, and reimbursement are aligned (Table 1).

**Table 1:** Conceptual summary of key outcomes and overall patterns in teledentistry

Main question addressed	General pattern and overall direction
Does teledentistry enhance access to care?	Consistent reports of reduced travel burden, improved reach to underserved populations, and more efficient triage pathways
How does remote assessment compare with in-person care?	Remote evaluation performs well for selected indications, particularly caries screening, orthodontic assessment, and triage of soft tissue lesions
How do patients perceive teledentistry?	High levels of satisfaction, convenience, and acceptance, especially when appointments replace travel or time off work
How do clinicians view teledentistry?	Generally positive attitudes toward access and triage benefits, with predictable concerns about training, workload, and medico-legal issues
Is teledentistry cost-efficient?	Demonstrates potential cost savings in settings involving long travel distances, school programs, and residential facilities
Can teledentistry support learning?	Effective for theoretical instruction, case-based learning, and remote supervision; limited for manual skill assessment

## Conclusions

Teledentistry has progressed to a practical component of routine oral healthcare in selected indications and settings. Current evidence suggests that, when appropriate technology and protocols are in place, remote consultations can support accurate diagnosis, safe triage, and acceptable follow-up for several clinical situations, particularly in orthodontics, pediatric dentistry, and community-based screening. The main, consistent advantages relate to improved access, reduced travel and waiting times, and high patient satisfaction. At the same time, the evidence base for long-term clinical outcomes, cost-effectiveness, and equity impact remains limited and heterogeneous. Teledentistry should therefore be implemented as part of planned hybrid care models, rather than as a stand-alone substitute for in-person dentistry. Future work needs to prioritize pragmatic trials and implementation studies that include robust outcome measures, economic evaluations, and equity indicators. If these conditions are met, teledentistry can contribute to more accessible, continuous, and patient-centered oral healthcare systems.
